# The Field of Science

**Published:** 1893-11-11

**Authors:** 


### The Field of Science.

The latest fashionable craze is the eucalyptus plant itself. This is now to be procured in a size convenient for the drawing-room, and is placed in a more or less magnificent câche-pôt, in as central a position as the furniture of the room permits. Here it is regarded, not only as a thing of beauty, but as a fetish to ward off infection from influenza and other contagious maladies.

Inter-hospital communication is greatly wanted in the metropolis. As matters stand at present, a patient requiring hospital treatment labours under the disadvantage of seeking this from the door of one institution to another, when, as has happened frequently, all available space is occupied previous to his arrival. Now, a few words down a tube would simplify this, and would save the sufferer many a needless ambulance journey. Such a remonstrance has found expression in the pages of a medical contemporary, and it is time that a salutary reform in the matter should take place. Why cannot all London hospitals be put into telephonic communication with one another? As it now stands, only three or four out of the total number of such institutions can be communicated with through this system. If it is want of means which hinders a furtherance of this project, so essential for the well-being of the community at large, then a special fund could surely be started with a view to meeting the difficulty, for so serious a want has only to be recognised to be met with substantial support from many quarters. It is only too true that the time is past when each hospital can be regarded as a self-conducted and self-contained entity; each, as has been said, is but part of the general hospital administration of the metropolis, and as such calls most emphatically for a closer intercommunication than is now existent.

English spelling, as evinced in American medical journals, is fast becoming a "foreign" language to the uninitiated British reader. To keep pace with these startling innovations (or omissions, to speak more technically), we now learn that the latest "improvements" up to date in our spelling will be incorporated in the second edition of the American Dictionary of Medical terms, which is shortly to appear. The English Press has not been wanting in raising protests against this most questionable etymological reform, but with little avail. Why do not Americans call this modern language after the land of its adoption, since it is fast losing all hold on the right to be named after the land of its birth, and then objections from this side of the water would cease.

It is not alone the female leaders of fashion in the Parisian capital who are to be held responsible for the increasing demand now existing for morphia; recent statistics show that the use of the drug is by no means confined to women of fashion. Indeed a large proportion of morphinomania in France is to be found among members of the medical profession, to whom, in the first instance, women owed their initiation into the deadly habit. Quite recently M. de Lecæsagne instituted some researches on this subject, which show on publication that of 545 cases which he came in contact with, no less than 289 were doctors. The evil is undoubtedly not only extending in new channels but startling advances are made in the number of victims to morphinomania in Paris. In 1883 the figures were put down to 40,000, but M. Debut de Laforet now estimates the number of persons succumbing to the overuse of the drug at 100,000.

